# Transcriptomic and Metabolomic Analyses Reveal the Differential Regulatory Mechanisms of Compound Material on the Responses of *Brassica campestris* to Saline and Alkaline Stresses

**DOI:** 10.3389/fpls.2022.820540

**Published:** 2022-02-23

**Authors:** Ziwei Li, Mengjie An, Dashuang Hong, Doudou Chang, Kaiyong Wang, Hua Fan

**Affiliations:** Agricultural College, Shihezi University, Shihezi, China

**Keywords:** forage crop, animal husbandry, saline–alkali land development, biosynthesis of other secondary metabolites, genetic adaptability

## Abstract

Oilseed rape not only has the function of improve saline and alkaline soils, but also alleviate the local feed shortage. However, medium- and high-degree soil salinization and alkalinization always inhibit the growth of oilseed rape. Studies have shown that compound material can improve the tolerance to saline and alkaline stress of crops, but the difference in the regulation mechanism of compound material on oilseed rape in saline and alkaline soils is not clear. This study explored the difference through determining the leaf ion contents, physiological indexes, transcriptomics, and metabolomics of oilseed rape in salinized soil (NaCl 8 g kg^−1^) and alkalinized soil (Na_2_CO_3_ 8 g kg^−1^) at full flowering stage, respectively after the application of compound material. The results showed that in salinized and alkalinized soil, the compound material upregulated the genes related to the regulation of potassium ion transport, and changed the amino acid metabolic pathway, which reduced the contents of Na^+^, malondialdehyde (MDA), and relative conductivity (REC) in leaves, and increased the contents of K^+^ and Mg^2+^ and the activities of superoxide dismutase (SOD), peroxidase (POD), and catalase (CAT). However, there were differences in the regulation mechanism of compound material in salinized and alkalinized soil. In salinized soil, the compound material improved the tolerance of oilseed rape to saline stress by upregulating transcription factors mannose-1-phosphate guanylyltransferase (*GPMM*) and Glutamine--fructose-6-phosphate transaminase (*GFPT*) and downregulating phosphomannomutase (*PMM*) to change nucleotide metabolism pathway and lipid metabolism pathway. In alkalized soil, the compound material improved the tolerance of oilseed rape to alkaline stress by upregulating transcription factors Phenylalanine ammonia lyase (*PAL*) to change the biosynthesis pathway of other secondary metabolites. Therefore, the compound material can improve the tolerance of oilseed rape to saline and alkaline stress by regulating the genetic adaptability and apparent plasticity, but the mechanisms were different. This study provides a practical method for the ecological environment restoration and the development of animal husbandry.

## Introduction

Soil salinization and alkalinization exert great pressures to the environment, and seriously threaten the sustainable development of agriculture. At present, there are about 8.31 × 10^8^ ha of land subjecting to varying degrees of salinization and alkalinization in the world (Cheeseman, 2013). In arid and semi-arid regions, soil salinization is characterized by high concentration of soluble salts, such as NaCl and Na_2_SO_4_, soil alkalinization is characterized by high concentration of Na_2_CO_3_ (Alvarez-Acosta et al., 2019). Excessive Na^+^ in salinized and alkalinized soil may cause ion toxicity to plants, thereby affecting the absorption of nutrients, mainly K^+^, by plants (Ma et al., 2019), resulting in plant wilting and death (Lu et al., 2021). There are also high concentrations of Ca^2+^, Mg^2+^, ${\mathrm{SO}}_{4}^{2-}$, and ${\mathrm{CO}}_{3}^{2-}$ ions in salinized soils that affect plant growth at the physiological and biochemical levels (Ma et al., 2019). Different from soil salinization, soil alkalinization could inhibit the absorption of some inorganic anions by plants, such as $H_{2}{\mathrm{PO}}_{4}^{-}$, Cl^−^, and ${\mathrm{NO}}_{3}^{-}$. The main component, Na_2_CO_3_, in alkalinized soil could affect the selective absorption of K^+^ and Na^+^ by plants, and destroy the ion balance, resulting in the hindered and abnormal growth of plant roots in soil (Yang et al., 2007). Therefore, exploring the differences in the mechanism of tolerance of plants to saline and alkaline stresses has guiding significance for the restoration and sustainable utilization of saline–alkali soils.

*Brassica campestris* can be used to alleviate the shortage of green fodder for cattle and sheep in arid and semi-arid regions in winter and spring (Wang et al., 2018). Besides, as a salt-tolerant crop, it also has the special advantage of utilizing and ameliorating saline–alkali soils. Wang (2020) found that planting *B. campestris* can reduce soil pH and Na^+^ content in saline–alkali soils, but Ulfat et al. (2020) have shown that medium and high degree of soil salinization and alkalinization could suppress the growth of *B. campestris* and reduce the yield. However, the application of modifiers, such as lignosulfonate, polyacrylamide, and polyglutamic acid, can reduce the damage of ionic toxicity, and change the physiological and biochemical indicators of the growth of *B. campestris* phenotype, such as the accumulation of proline and the increase of T-AOC (Xu et al., 2017), synthesis of lignin and fatty acids, alleviating oxidative stress, and regulating the osmotic pressure of cells (Chen, 2020; Huang, 2021), thereby improving the ability of crops to resist saline and alkaline stresses (Ahmad, 2012; Wang et al., 2021). Therefore, the application of modifiers combined with the cultivation of salt-tolerant forage crop to ameliorate saline–alkali soils provides a new way for the development of animal husbandry with saline–alkali soil resources.

Many studies have clarified that modifiers can regulate the genetic adaptation of crops in saline–alkali soils to improve the tolerance to saline and alkaline stresses. Tan et al. (2019) have found that melatonin could regulate the hormone synthesis and lignin and fatty acid metabolism in leaves and root of rape seedlings, which regulates the production of phytohormones and promotes their functions, thereby increasing the biomass of *B. campestris*. Mir et al. (2018) have found that jasmonic acid could regulate the proline and glutathione metabolic pathways of plant cells and improve the redox state of plants under alkaline stress. The compound materials including lignosulfonate and polyacrylamide can upregulate K^+^ transporter gene and K^+^/Na^+^ ratio in plant leaves and regulate the osmotic pressure and antioxidant system under saline and alkaline stresses by upregulating phenylpropane biosynthesis pathway (An et al., 2020).

However, there are few studies on the differences in regulation performance of genetic adaptation of *B. campestris* to saline and alkaline stresses by compound materials. Therefore, the purpose of this study was to explore the regulatory mechanisms of compound material on the tolerance of *B. campestris* to saline and alkaline stresses from the perspectives of phenotypic plasticity and genetic adaptability through field experiments. This study determined (1) the differences in the physiological characteristics of *B. campestris* under saline stress and alkaline stress, (2) the molecular regulation mechanism of CM on the responses of *B. campestris* to saline stress and alkaline stress, and (3) the difference between the self-regulation mechanism of *B. campestris* in response to saline and alkaline stresses and the regulation mechanism of compound material. Our study could reveal the differential regulation mechanism of CM on the responses of *B. campestris* to saline stress and alkaline stress, and help develop a practical method for sustainable development of agriculture in saline and alkaline soils.

## Materials and Methods

### Experimental Location and Experimental Materials

This experiment was conducted at Shihezi, Xinjiang, China (44°33′46.1″N, 86°02′81″E) from July 15 to October 20, 2020. The soil is a grey desert soil. Soil properties are shown in Table 1 (Manoj et al., 2020). *B. campestris* seeds (variety Huayouza 82) were obtained from Huazhong Agricultural University (Wuhan, China). CM was prepared through mixing polyacrylamide, polyvinyl alcohol, manganese sulfate, and inorganic fertilizer (superphosphate of 180 kg/ha and urea of 240 kg/ha) at the mass ratio of 3: 1: 6: 50.

**Table 1:** Physicochemical properties of soil.

Item	Value
pH	8.25
Cation exchange capacity (CEC)	17.32 coml kg^−1^
Organic matter content	12.5 g kg^−1^
Alkali-hydrolyzable nitrogen content	54 mg kg^−1^
Available phosphorus content	11.7 mg kg^−1^
Available potassium content	218 mg kg^−1^

### Experimental Design

This experiment employed a randomized block design as six plant per block, with a total of four treatments. Each treatment had three replicates. The four treatments were (1) YP treatment (NaCl of 8 g kg^−1^ was mixed fully with the plough layer (0–20 cm) and CM of 12 kg hm^−2^ was applied), (2) JP treatment (Na_2_CO_3_ of 8 g kg^−1^ was mixed fully with the plough layer and CM of 12 kg hm^−2^ was applied), (3) YCK treatment (no CM was applied and NaCl of 8 g kg^−1^ was mixed fully with the plough layer), and (4) JCK treatment (no CM was applied and Na_2_CO_3_ of 8 g kg^−1^ was mixed fully with the plough layer). On July 20, 2020, soils were put in plastic buckets (length × width × height: 30 × 30 × 80 cm) while maintaining the original profile of the soil layer, and buried in the field, followed by the application of NaCl and Na_2_CO_3_ separately. The pH and EC of salinized soil were 9.12 and 3.52 s m^−1^, respectively, and those of alkalinized soil were 9.59 and 3.85 s m^−1^, respectively. *B. campestris* seeds were sown through mixing with fertilizers (superphosphate of 180 kg/ha, urea of 240 kg/ha) at a ratio of 1:15 on July 15th. The CM was applied through drip irrigation after diluted with water. Drip irrigation (300 m^3^/ha) was started in the seedling stage, with a cycle of 10 days until the full flowering stage. Leaves were collected in the full flowering stage (on October 20th) and immediately placed in liquid nitrogen and stored at −80°C until use. All measurements were repeated three times, and the average value was used for analysis.

### Leaf Physiological Analysis

The leaves, stems and roots of *B. campestris* were separated, deactivated in an oven at 105°C for 30 min, dried at 70°C, and weighed. The accumulation of dry matter for each organ was measured (Bao, 2000). The content of Na^+^ and K^+^ in the leaves was measured according to the method of Bao (2000). Leaf samples were immersed in the mixture of 98% H_2_SO_4_ and 30% H_2_O_2_, and measured with an AP1200 flame spectrophotometer (AP1200 type, Shanghai, China). The content of Ca^2+^ and Mg^2+^ was measured according to the EDTA titration method (Goffman et al., 2004), the content of ${\mathrm{SO}}_{4}^{2-}$ and Cl^−^ was measured according to the EDTA indirect complexometric titration method and the silver nitrate titration method, respectively, and the content of ${\mathrm{CO}}_{3}^{2-}$ and ${\mathrm{HCO}}_{3}^{-}$ was measured according to the dual-indicator neutralization titration method (Moustaka et al., 2016). The activity of antioxidant enzymes in leaves (0.5 g) was measured by spectrophotometry (Ashraf and Ali, 2008). The SOD activity was measured at 560 nm based on NBT photochemical reduction. The peroxidase (POD) activity was measured at 470 nm based on the absorbance of guaiacol. The CAT activity was measured at 240 nm by reacting potassium phosphate buffer with H_2_O_2_. The content of malondialdehyde (MDA) was measured according to the content of thiobarbituric acid reactive substances (TBARS) in leaf samples (nmol/g; extinction coefficient: 155 mM cm^−1^; Wang et al., 2017). Fresh leaves (0.1 g) were cut into 1 cm slices, put in 10 ml of deionized water, and shaken on a rotary shaker for 24 h at room temperature to measure the electrical conductivity (L_1_) using a conductivity meter (EM38, ICT international, Armidale, NSW, Australia). After that, the solution was boiled for 15 min, and the electrical conductivity of the solution was measured again (L_2_). Finally, the relative electrical conductivity (REC) was calculated according to the following formula (Ding et al., 2018):


$$REC=L_{1}/L_{2}$$

### Transcriptome Sequencing and Data Analysis

The total RNA of 12 samples were processed for transcriptome sequencing, and qualified RNA were processed for library construction. mRNA was isolated by Oligo(dT)-attached magnetic beads, and then randomly fragmented in fragmentation buffer. First-strand cDNA was synthesized with fragmented mRNA as template and random hexamers as primers, followed by second-strand synthesis with addition of PCR buffer, dNTPs, RNase H, and DNA polymerase I. Purification of cDNA was processed with AMPure XP beads (Zhuang et al., 2019). Double-strand cDNA was subjected to end repair. Adenosine was added to the end and ligated to adapters. AMPure XP beads were applied here to select fragments within the size range of 300–400 bp. cDNA library was obtained by certain rounds of PCR on cDNA fragments generated from step 4.

Qubit 2.0 and Agilent 2,100 were used to examine the concentration of cDNA and insert size, and Q-PCR was performed to obtain a more accurate library concentration, to ensure the quality of library. Library with concentration higher than 2 nM was acceptable. The qualified library was pooled based on pre-designed target data volume and then sequenced on Illumina sequencing platform (Liu et al., 2020). Clean data with high quality was obtained through filtering raw data to remove adapter sequence and reads with low quality. These clean data were further mapped to pre-defined reference genome to obtain mapped data. Assessment on insert size and sequencing randomness were performed on mapped data as library quality control. Basic analysis on mapped data included gene expression quantification, alternative splicing analysis, novel genes prediction, and genes structure optimization. Data analysis was performed using BMKCloud.^1^

### Metabolomics and Data Analysis

Metabolite analysis was performed on 12 samples using the non-target metabolomics profiling (Sun et al., 2021). Frozen leaf sample (200 mg) was added with 1,000 μl of solution consisted of methanol, acetonitrile, and water (V: V: V = 2:2:1, 2 μg/ml) and vortexed for 30 s. After that, ceramic beads were added, and processed with a grinder (45 Hz) for 4 min, following by the ultrasonication in an ice-water bath for 5 min. This step was repeated for 3 times. After centrifugation at 12,000 rpm for 15 min, the supernatant was removed and placed in an EP tube. The extract was dried in a vacuum concentrator. Then, 150 μl of solution consisted of acetonitrile and water (V: V = 1:1) was added to the dried metabolites, vortexed for 30 s, and ultrasonicated in an ice-water bath for 10 min. The sample was centrifuged at 12000 rpm at 4°C for 15 min. The supernatant (120 μl) was transferred into a 2 ml sample bottle, and 10 μl from each bottle was mixed for testing (Zhang et al., 2015).

The LC/MS system for metabolomics analysis was composed of Waters Acquity I-Class PLUS Ultra Performance Liquid Chromatography in tandem with Waters Xevo G2-XS QT of High Resolution Mass Spectrometer. The Waters Acquity UPLC HSS T3 chromatography column (1.8 μm 2.1*100 mm) was used. The positive ion mode employed mobile phase A: 0.1% formic acid aqueous solution and mobile phase B: 0.1% formic acid acetonitrile. The negative ion mode employed mobile phase A: 0.1% formic acid aqueous solution and mobile phase B: 0.1% formic acid acetonitrile. The injection volume was 1–3 μl. The gradient elution procedures were 0–0.25 min 98% A, 0.25–10 min 2% A, 10–13 min 2% A, 13–13.1 min 98% A, and 13.1–15 min 98% A. The flow rate was 400 μl/min. The raw data collected using MassLynx V4.2 were processed by using Progenesis QI software (version 3.0) for peak extraction, peak alignment, etc. Online databases, such as METLIN and Biomark’s self-built libraries, were used for identification based on the type of samples using the Progenesis QI software. The mass deviation of fragment ion identification was within 100 ppm. The analysis of metabolome data was performed on the platform BMKCloud (see footnote 1).

### Statistical Analysis

One-way ANOVA analysis and Duncan test of the ion content and physiological characteristics of *B. campestris* leaves were performed using SPSS 22.0 software (SPSS, Chicago, IL, United States) at *p* < 0.05. R software (version 3.6.1; Boston, MA, United States)^3^ was used to process the data. Origin software (version 2018; Origin Laboratories, Ltd., Northampton City, MA, United States) and Gephi software (version 0.9.2)^4^ were used for drawing.

## Results

### Phenotype Analysis of *B. campestris* Leaves

#### Dry Matter of *B. campestris* Leaves

The growth of *B. campestris* was obviously better in NaCl treatments (YCK and YP treatment) than in Na_2_CO_3_ treatment (JCK and JP treatment). The application of CM obviously increased the dry weight of *B. campestris* organs in all treatments. For instance, the dry weight of root, stem, and leaves in YP treatment were 41, 33, and 59% higher than those in YCK treatment, respectively (*p* < 0.05), and the dry weight of root, stem, and leaves in JP treatment were 33, 51, and 59% higher than those in JCK treatment, respectively (*p* < 0.05). The dry weight of leaves was lower in JCK treatment than in YCK treatment. However, there was no difference in the dry weight of stem (*p* > 0.05), but the root dry weight was higher in JCK treatment than in YCK treatment (Table 2).

**Table 2:** Dry weight of *Brassica campestris* organs.

Parameters	YCK	YP	JCK	JP
Root dry weight (g)	9.01 ± 0.72b	12.7 ± 1.11a	9.89 ± 1.33b	13.10 ± 0.85a
Stem dry weight (g)	40.43 ± 2.89b	58.99 ± 6.95a	39.69 ± 2.58b	59.87 ± 5.22a
Leaf dry weight (g)	18.04 ± 1.96b	28.77 ± 1.70a	16.56 ± 2.03b	26.37 ± 0.88a

*Different lowercase letters after the data indicate significant difference at p < 0.05*.

#### Ion Content and Physiological Characteristics of *B. campestris* Leaves

**Figure 1:**
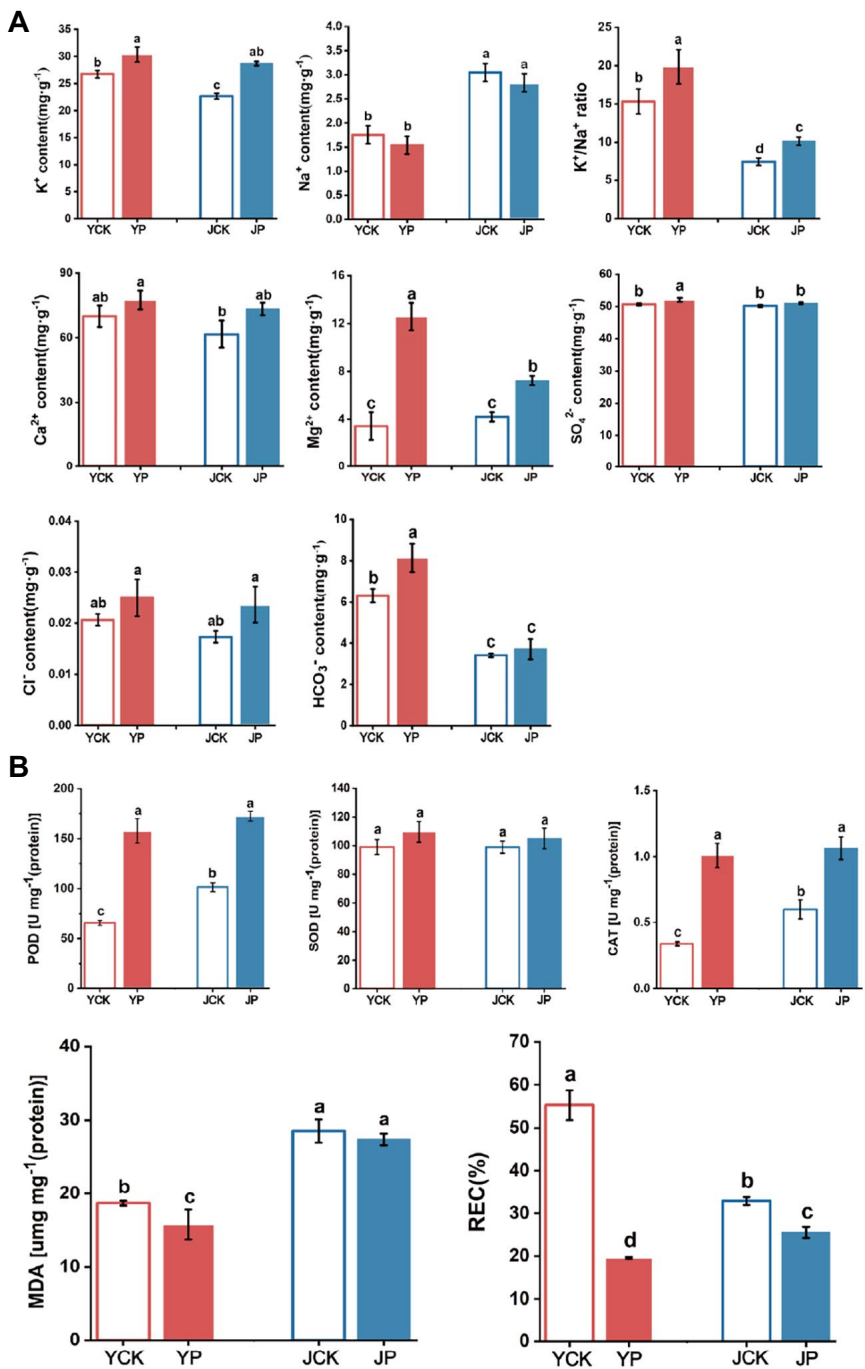
Changes in ion content (K^+^, Na^+^, K^+^/Na^+^ ratio, Ca^2+^, Mg^2+^, ${\mathrm{SO}}_{4}^{2-}$, Cl^−^, and ${\mathrm{HCO}}_{3}^{-}$) in *Brassica campestris* leaves in different treatments **(A)**, and changes in antioxidant enzymes activity and the content of MDA and REC in *Brassica campestris* leaves in different treatments **(B)**. Different lowercase letters indicate significant difference at *p* < 0.05.

The content of K^+^ and REC in leaves in Na_2_CO_3_ treatment (JCK and JP treatment) were lower than those in NaCl treatments (YCK and YP treatment; *p* < 0.05), while the activities of POD and CAT and the content of MDA were higher than those in NaCl treatments (*p* < 0.05). The application of CM increased the content of K^+^ and Mg^2+^, K^+^/Na^+^ ratio, and the activity of POD and CAT, while reduced the content of Na^+^ and REC. The content of Na^+^ in leaves was higher in Na_2_CO_3_ treatments (JCK and JP treatment) than in NaCl treatments (YCK and YP treatment). There was no difference in Na^+^ content between YP treatment and YCK treatment (*p* > 0.05). However, the content of K^+^, Mg^2+^, ${\mathrm{HCO}}_{3}^{-}$ and K^+^/Na^+^ ratio in YP treatment was 14, 266, 29, and 30% higher than those in YCK treatment, respectively (*p* < 0.05). There was also no difference in Na^+^ content between JP treatment and JCK treatment (*p* > 0.05). However, the content of K^+^ and Mg^2+^ and K^+^/Na^+^ ratio in JP treatment were 18% (*p* < 0.05), 73% (*p* < 0.05), and 36% (*p* > 0.05) higher than those in JCK treatment, respectively. The content of ${\mathrm{SO}}_{4}^{2-}$ had no difference among treatments. The activity of SOD and POD in JCK treatment was 2.3% (*p* > 0.05) and 54% (*p* < 0.05) higher than those in YCK treatment, respectively, while the activity of CAT was 29% lower than that in YCK treatment. The activity of SOD and POD in YP and JP treatment was higher than those in the control (YCK and JCK treatment). The activity of SOD and POD in the YP treatment was 8.6% (*p* > 0.05) and 140.17% (*p* < 0.05) higher than those in the YCK treatment, respectively, while the activity of CAT, MDA, and REC were 85, 15, and 65% lower than those in the YCK treatment, respectively (*p* < 0.05). The activities of CAT and POD in the JP treatment were 70 and 129% higher than those in the JCK treatment, respectively (*p* < 0.05), while the content of MDA and REC were 7.2 and 22% lower than those in the JCK treatment, respectively (*p* < 0.05). There was no difference in SOD activity between JP treatment and JCK treatment (*p* > 0.05; Figure 1).

#### Transcriptome Analysis for *B. campestris* Leaves

**Figure 2:**
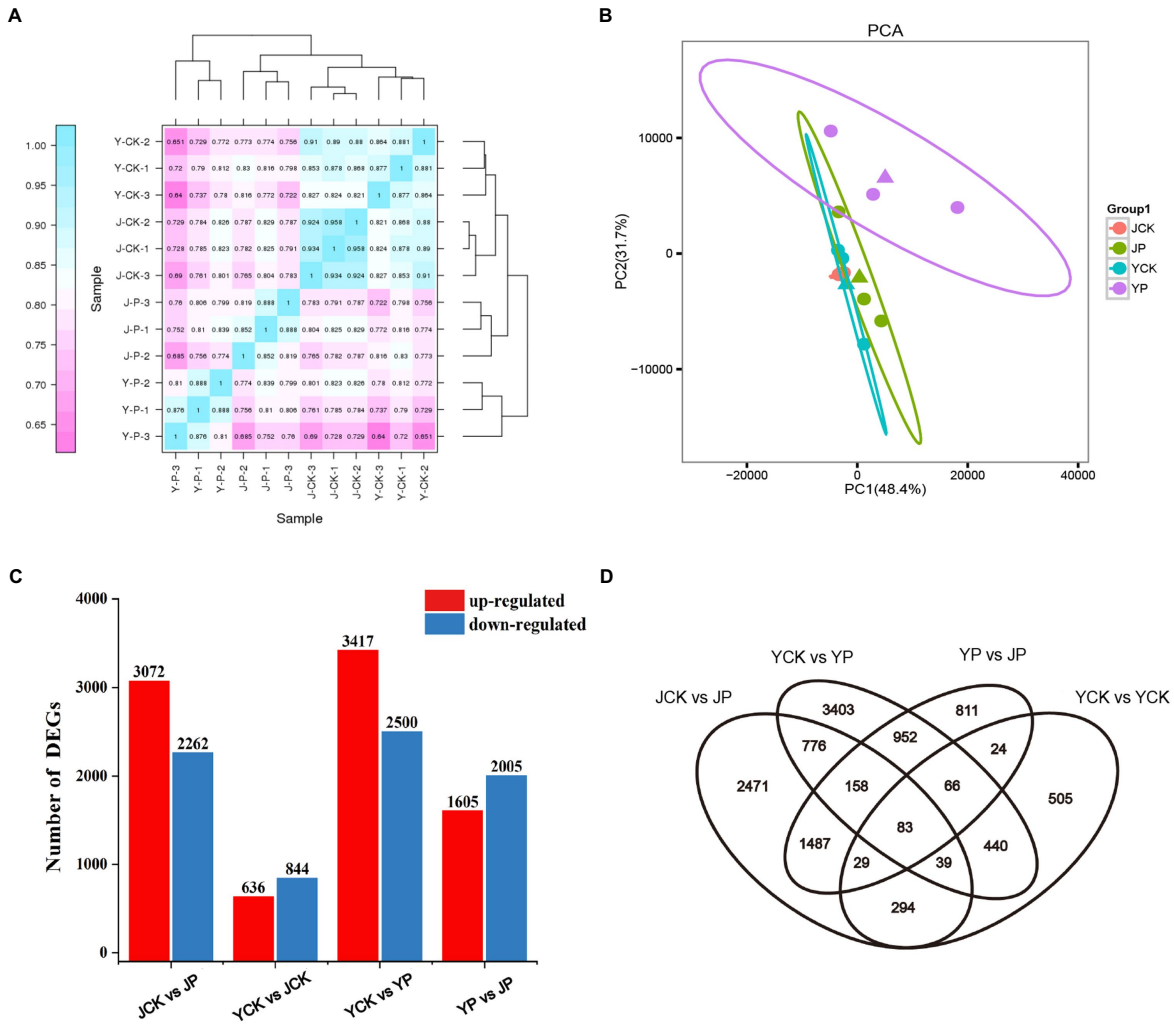
Heatmap of correlations among samples in different treatments **(A)**, PCA analysis **(B)**, the number of DEGs in leaves identified in different treatments by transcriptomic analysis **(C)**, and Venn diagram of DEGs **(D)**.

High throughput sequencing of *B. campestris* leaves was performed by using Illumina Hiseq platform, and the average reading times was 22 million. The clean reads of each sample were compared with the specified reference genome, and the efficiency ranged from 81 to 91%. The counts of reads were summarized at the gene level (Supplementary Table S1). The heatmap proved that the samples had good repeatability (Figure 2A). Principal component analysis (PCA) showed that the samples in NaCl and Na_2_CO_3_ treatments were obviously separated in PC2 dimension, while the samples in compound material treatments and the controls were obviously separated in PC1 dimension (Figure 2B). To determine the difference of transcriptional response of different treatments, differentially expressed genes (DEGs) were determined by pairwise comparison of samples. Compared to YCK treatment, the expression of 636 genes was upregulated and that of 844 genes were downregulated in JCK treatment (Figure 2C). A total of 5,337 and 5,917 DEGs were identified in Na_2_CO_3_ treatments (JCK and JP treatment) and NaCl treatments (YCK and YP treatment), respectively (Figure 2C). Compared to YCK treatment, the expression of 3,417 genes was upregulated and that of 2,500 genes were downregulated in YP treatment. Compared to JCK treatment, the expression of 3,072 genes was upregulated and that of 2,262 genes were downregulated in JP treatment (Figure 2C). Compared to YP treatment, the expression of 1,605 genes was upregulated and that of 2005 genes were downregulated in the JP treatment. The Venn diagram showed that there was a total of 83 common DEGs for the four treatments (Figure 2D).

**Figure 3:**
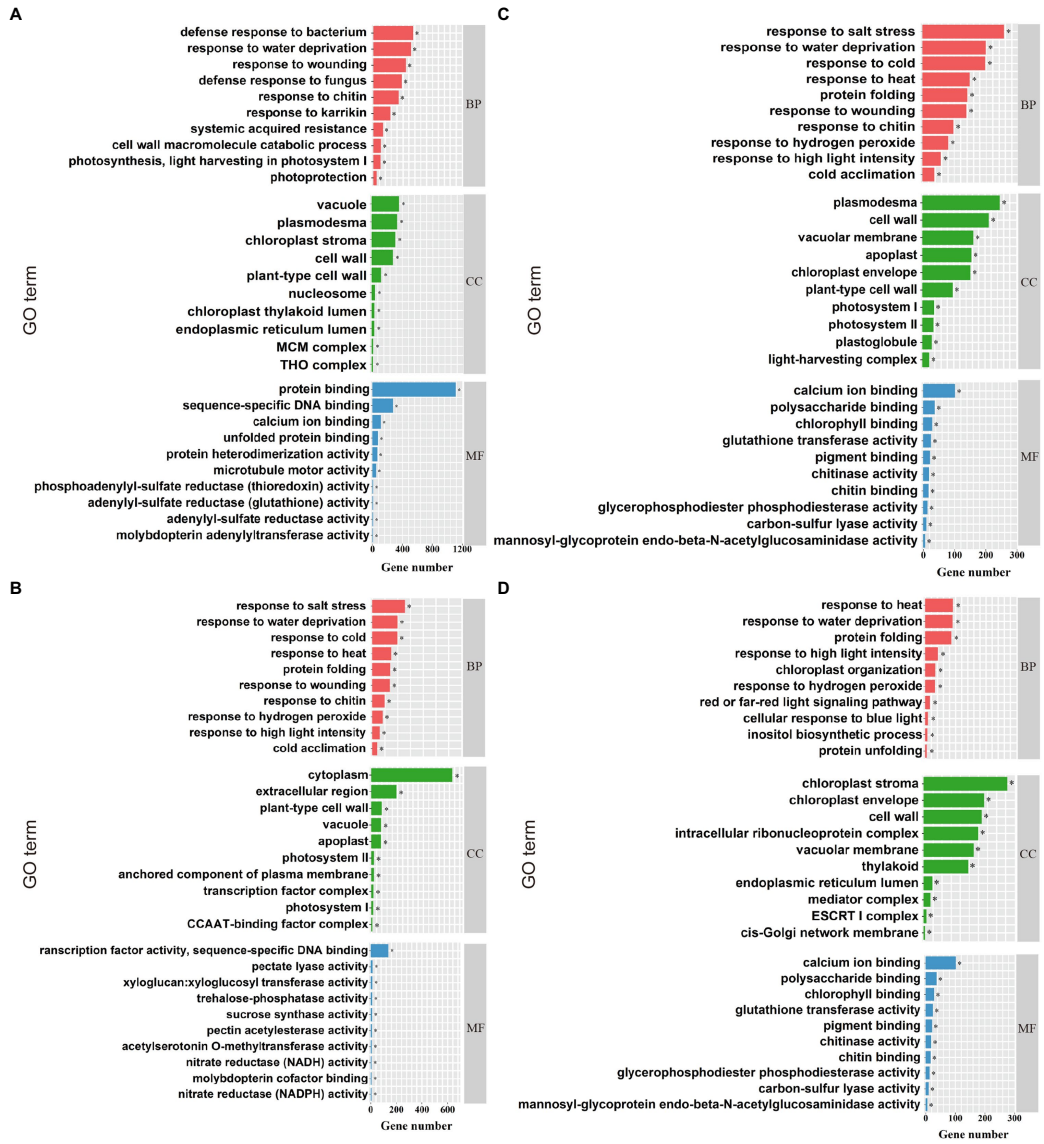
GO enrichment analysis. The top ten enriched GO terms for NaCl treatments (YCK and YP treatment; **A**), the top ten enriched GO terms for Na_2_CO_3_ treatments (JCK and JP treatment; **B**), the top ten enriched GO terms for the control (YCK and JCK treatment; **C**), and the top ten enriched GO terms for compound material treatments (YP and JP treatment; **D**). BP, CC, and MF represent biological process, cellular component, and molecular function, respectively. *Indicates significant difference at *p* < 0.05.

Gene ontology (GO) enrichment analysis was performed on DEGs in compound material treatments (YP and JP treatment), and three functions [biological process (BP), cellular component (CC), and molecular function (MF)] were annotated. For NaCl treatments (YCK and YP treatment; Figure 3A), the most enriched GO terms in BP were response to salt stress (GO: 0009651), response to water deprivation (GO: 0009414), and response to cold (GO: 0009409). The most enriched GO terms in CC were chloroplast stroma (GO: 0009570), vacuole (GO: 0005773), and plasma (GO: 0009506). The most enriched GO terms in MF were protein binding (GO: 0005515), sequence-specific DNA binding (GO: 0003700), and calcium ion binding (GO: 0043565). For Na_2_CO_3_ treatments (JCK and JP treatment; Figure 3B), the most enriched GO terms in BP were defense response to bacterium (GO:0042742), response to water deprivation (GO:0009414), and response to wounding (GO:0009611). The most enriched GO terms in CC were plasmodesma (GO:0000786), cell wall (GO:0042555), and vacuolar membrane (GO:0005874). The most enriched GO terms in MF were calcium ion binding (GO:0005509), polysaccharide binding (GO:0030247), and chlorophyll binding (GO:0016168). For the control (YCK and JCK treatment; Figure 3C), the most enriched GO terms in BP were response to far red light (GO:0010218), pectin catabolic process (GO:0045490), and nitrate assimilation (GO:0042128). The most enriched GO terms in CC were cytoplasm (GO:0005737), extracellular region (GO:0005576), and plant-type cell wall (GO:0009505). The most enriched GO terms in MF were transcription factor activity sequence-specific DNA binding (GO:0003700), pectate lyase activity (GO: 0030570), and xyloglucan: xyloglucosyl transferase activity (GO:0016762). For compound material treatments (YP and JP treatment; Figure 3D), the most enriched GO terms in BP were response to heat (GO:0034605), response to water deprivation (GO: 0009414), and protein folding (GO:0006457). The most enriched GO terms in CC were chloroplast stroma (GO:0009570), chloroplast envelope (GO:0009941), and cell wall (GO:0005618). The most enriched GO terms in CC were ATPase activity (GO:0016887), unfolded protein binding (GO:0051082), and thioglucosidase activity (GO:0019137).

Kyoto encyclopedia of genes and genomes (KEGG) enrichment analysis (Supplementary Figure S1) helps us further understand the molecular interactions among DEGs. The results showed that the CM regulated the complex biological pathways in *B. campestris* under saline and alkaline stresses. For NaCl treatments (YCK and YP treatment), DEGs were obviously enriched in the pathways of protein processing in endoplasmic reticulum, plant–pathogen interaction, amino sugar and nucleotide sugar metabolism, and DNA replication. For Na_2_CO_3_ treatments (JCK and JP treatment), DEGs were obviously enriched in the pathways of plant–pathogen interaction, phenylpropanoid biosynthesis, and amino sugar and nucleotide sugar metabolism. For the control (YCK and JCK treatment), DEGs were obviously enriched in the pathways of starch and sucrose metabolism, phenylpropanoid biosynthesis, pentose and glucuronate interconversions, and circadian rhythm plant. For compound material treatments (YP and JP treatment), DEGs were obviously enriched in the pathways of protein processing in endoplasmic reticulum, spliceosome, and plant–pathogen interaction.

#### Regulation of Responses of Antioxidant Activity and Salt Ion Transporter in *B. campestris* Leaves

Many DEGs identified in *B. campestris* leaves were enriched in GO terms related to oxidoreductase (Table 3) and ion transport (Table 4). For instance, in NaCl treatments, the expression of four genes related to peroxidase activity (*BnaA01G0372800ZS*, *BnaA10G0269200ZS*, *BnaC01G0465300ZS*, and *BnaC05G0022700ZS*), three genes related to potassium ion transport (*BnaA08G0067600ZS*, *BnaC05G0326000ZS,* and *BnaC08G0094500ZS*), and three genes related to the regulation of potassium ion transport (*BnaA09G0714900ZS*, *BnaA10G0007700ZS*, and *BnaC05G0009300ZS*) were obviously upregulated. In Na_2_CO_3_ treatments, the expression of three superoxide dismutase genes (*BnaA09G0129500ZS*, *BnaA09G0647100ZS*, and *BnaC09G0137500ZS*) and three genes related to potassium ion transport (*BnaA05G0313200ZS*, *BnaC06G0097600ZS,* and *Bnascaffold0075G0000100ZS*) were upregulated.

**Table 3:** Expression patterns of DEGs involved in ROS.

	ID	log2FC	Up/down	*p*-value	Padj	Description
JP-JCK	BnaA09G0129500ZS	2.201133591	Up	0.000712	0.023299	Superoxide dismutase copper chaperone activity
	BnaA09G0647100ZS	1.960363331	Up	0.000712	0.023299	Superoxide dismutase copper chaperone activity
	BnaC09G0137500ZS	3.148717758	Up	0.000712	0.023299	Superoxide dismutase copper chaperone activity
YP-YCK	BnaA01G0372800ZS	−2.278459345	Down	9.54E-05	0.004568	Peroxiredoxin activity
	BnaA10G0269200ZS	−1.657701649	Down	9.54E-05	0.004568	Peroxiredoxin activity
	BnaC01G0465300ZS	−1.701344159	Down	9.54E-05	0.004568	peroxiredoxin activity
	BnaC05G0022700ZS	−5.226903086	Down	9.54E-05	0.004568	Peroxiredoxin activity
JCK-YCK	BnaA06G0161600ZS	−1.506716999	Down	0.018744	0.056788	Peroxidase activity
	BnaA07G0326900ZS	−2.750301758	Down	0.018744	0.056788	Peroxidase activity
	BnaC05G0035900ZS	−1.689632711	Down	0.018744	0.056788	Peroxidase activity
	BnaC05G0537900ZS	−1.907631862	Down	0.018744	0.056788	Peroxidase activity
	BnaC06G0382700ZS	−1.516621483	Down	0.018744	0.056788	Peroxidase activity
JP-YP	BnaA10G0269200ZS	1.644640075	Up	6.03E-05	0.008337	Peroxiredoxin activity
	BnaC01G0465300ZS	1.699869986	Up	6.03E-05	0.008337	Peroxiredoxin activity
	BnaC04G0340500ZS	3.398139077	Up	6.03E-05	0.008337	Peroxiredoxin activity
	BnaC06G0326200ZS	5.060488968	Up	6.03E-05	0.008337	Peroxiredoxin activity

**Table 4:** Expression patterns of DEGs involved in salt ion transport.

	ID	log2FC	Up/down	*p*-value	Padj	Description
JP-JCK	BnaA05G0313200ZS	2.364280808	Up	0.002052	0.024353	Potassium ion export
	BnaC06G0097600ZS	5.330866753	Up	0.002052	0.024353	Potassium ion export
	Bnascaffold0075G0000100ZS	2.568542631	Up	0.002052	0.024353	Potassium ion export
YP-YCK	BnaA09G0714900ZS	1.65256347	Up	3.56E-05	0.000419	Regulation of potassium ion transport
	BnaA10G0007700ZS	1.666847884	Up	3.56E-05	0.000419	Regulation of potassium ion transport
	BnaC05G0009300ZS	1.816344603	Up	0.0000356	0.000419	Regulation of potassium ion transport
	BnaA08G0067600ZS	2.593684063	Up	0.003059	0.016765	Potassium ion export
	BnaC05G0326000ZS	1.999559697	Up	0.003059	0.016765	Potassium ion export
	BnaC08G0094500ZS	2.523855231	Up	0.003059	0.016765	Potassium ion export
JP-YP	BnaC03G0308500ZS	3.195992075	Up	7.37E-03	0.167847	Sodium ion transport
	BnaA02G0187200ZS	−1.50476608	Down	2.74E-03	0.033256	Potassium ion transmembrane transport
	BnaA09G0699100ZS	1.963472502	Down	2.74E-03	0.033256	Potassium ion transmembrane transport
	BnaC01G0129800ZS	1.842244827	Down	0.002742	0.033256	Potassium ion transmembrane transport
	BnaC01G0494100ZS	6.399754121	Down	0.002742	0.033256	Potassium ion transmembrane transport
	BnaC02G0497000ZS	2.181474533	Down	0.002742	0.033256	Potassium ion transmembrane transport
	BnaA01G0087300ZS	3.179790725	Down	0.009008	0.071687	Potassium ion import
	BnaC07G0416200ZS	2.270572634	Down	0.009008	0.071687	Potassium ion import
	BnaA01G0219900ZS	2.947017252	Down	0.005989	0.091784	Potassium ion binding
	BnaA10G0095200ZS	4.371192662	Down	5.99E-03	0.091784	Potassium ion binding
	BnaC01G0279500ZS	2.906459361	Down	5.99E-03	0.091784	Potassium ion binding

#### Metabolome Analysis of *B. campestris* Leaves

**Figure 4:**
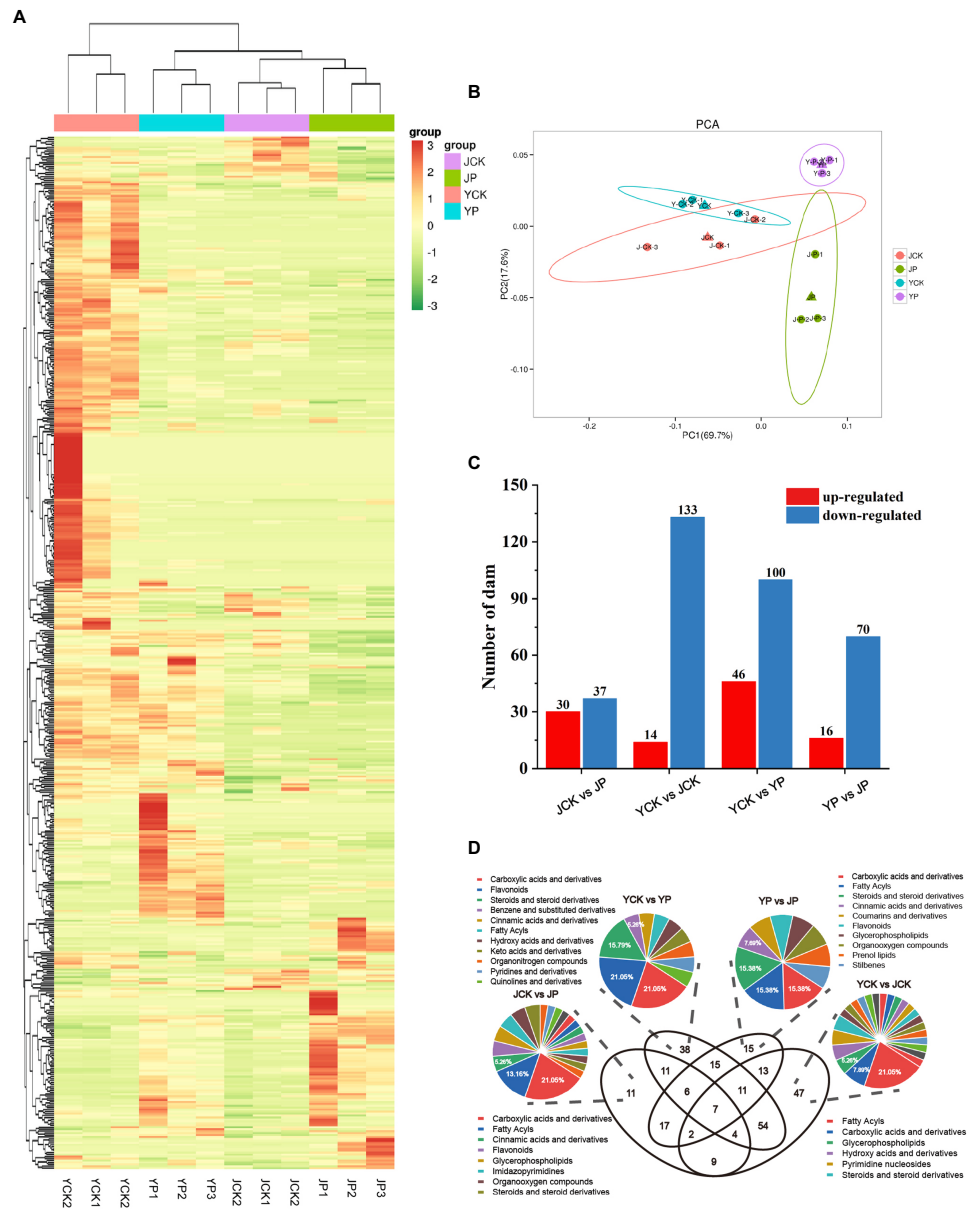
Analysis of metabolites of *Brassica campestris* in different treatments. **(A)** Heat map of metabolites. The content of each metabolite was normalized to complete linkage hierarchical clustering. Each treatment was shown in a column, and each metabolite was represented by a row. Red indicates high abundance, and green indicates low abundance. **(B)** Principal component analysis of metabolites. **(C)** The number of DEMs upregulated and downregulated in different treatments. **(D)** Venn diagram of DEMs and classification.

To further explore the mechanism of CM in improving the tolerance to saline and alkaline stress in *B. campestris* leaves, the differentially expressed metabolites (DEMs) were identified by high-performance liquid chromatography. A total of 533 metabolites were identified based on the widely targeted metabolomics (Figure 4A). The PCA results showed that there was an obvious separation among treatments, in which the samples in NaCl and Na_2_CO_3_ treatments were obviously separated in PC2 dimension, while the samples in CM treatments and the control were obviously separated in PC1 dimension (Figure 4B). A total of 260 DEMs were identified through pairwise comparison of samples. Compared to YCK treatment, the expression of 14 metabolites was upregulated and that of 133 metabolites were downregulated in JCK treatment (Figure 4C). The number of metabolites with expression changed was more in NaCl treatments than in Na_2_CO_3_ treatments. Compared to YCK treatment, the expression of 46 metabolites was upregulated and that of 100 metabolites were downregulated in YP treatment. Compared to JCK treatment, the expression of 30 metabolites was upregulated and that of 37 metabolites were downregulated in JP treatment (Figure 4C). Compared to YP treatment, the expression of 16 metabolites was upregulated and that of 70 metabolites were downregulated in JP treatment. Venn diagram showed that there were seven common DEMs for the four treatments (Figure 4D). The DEMs were annotated and classified. The results showed that four carboxylic acids and derivatives and four flavonoids were annotated for the DEMs in NaCl treatments. Eight carboxylic acids and derivatives and five fatty acyls were annotated for the DEMs in Na_2_CO_3_ treatments. Eight fatty acyls and four carboxylic acids and derivatives were annotated for the DEMs in the control. Two carboxylic acids and derivatives and two fatty acyls were annotated for the DEMs in CM treatments (Figure 4D).

#### Integrated Transcriptomic and Metabolomic Analyses in *B. campestris* Leaves

**Figure 5:**
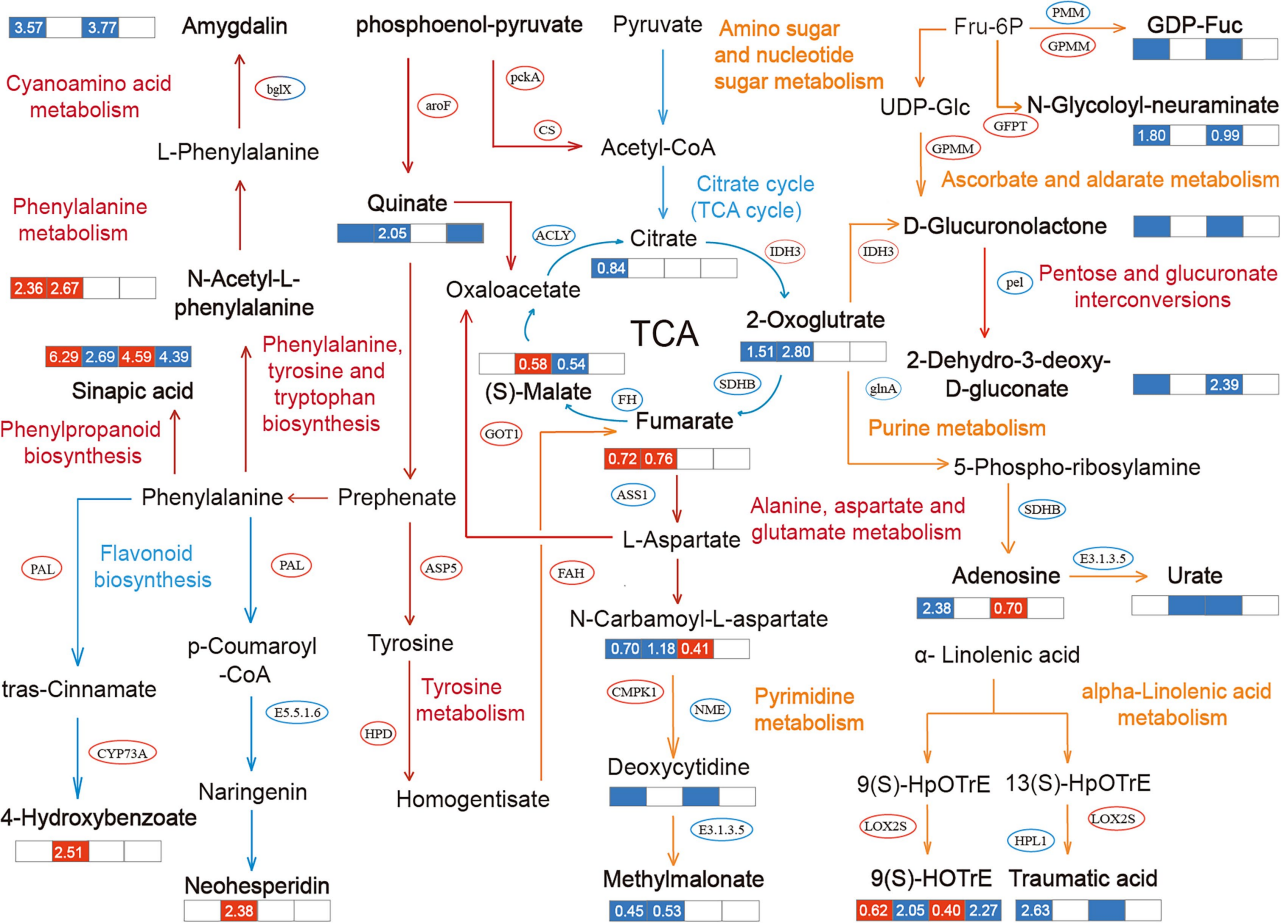
Metabolic pathways and the mutual regulation relationships in different treatments. The pathway notes and arrows in different colors represent the regulation pathways for different treatments. Red represents the common regulation pathway of NaCl treatments (YCK and YP treatment) and Na_2_CO_3_ treatments (JCK and JP treatment), yellow represents the regulation pathway of NaCl treatments (YCK and YP treatment), and blue represents the regulation pathway of Na_2_CO_3_ treatments (JCK and JP treatment). The ellipse indicates the obviously changed transcription factors, and the bold font indicates the differentially expressed metabolites (DEMs). The data in the box represents the average expression of metabolites for the two treatments (log2FC), in which red indicates upregulation, and blue indicates downregulation.

The association between metabolites and genes in *B. campestris* leaves in the same biological process was analyzed by KEGG annotation (Supplementary Figure S2). The pathways obviously enriched for different treatments were marked through integrated transcriptomic and metabolomic analyses (Figure 5). It was found that the pathways related to carbohydrate metabolism and amino acid metabolism were obviously enriched for the treatments. Among them, the DEGs and DEMs in NaCl treatments were enriched in the pathways of carbohydrate metabolism, nucleotide metabolism, and amino acid metabolism. The DEGs and DEMs in Na_2_CO_3_ treatments were enriched in the pathways of amino acid metabolism and biosynthesis of other secondary metabolites. The DEGs and DEMs in the control and CM treatments were mainly enriched in the pathway of amino acid metabolism.

Different regulatory mechanisms were found in the control (YCK and JCK treatment). Compared to YCK treatment, the expression of 2-dehydro-3-deoxy-d-gluconate was downregulated by downregulating the expression of pel in pentose and glucuronate interconversions pathway in JCK treatment. At the same time, the expression of 4-hydroxybenzoic acid was downregulated by upregulating the expression of PAL and CYP73A in the phenylalanine metabolism pathway. The expression of 3-amino-3-(4-hydroxyphenyl) propanoate in the tyrosine metabolism pathway was downregulated by 0.94 times, and that of amygdalin was downregulated by 3.77 times through the regulation of the expression of bglx in the cyanoamino acid metabolism pathway. After the application of CM, the regulation mechanisms of the responses of *B. campestris* to saline and alkaline stresses were only manifested in the pathway of phenylalanine, tyrosine, and tryptophan biosynthesis. Compared to YP treatment, the expression of Quinate in JP treatment was downregulated by 1.26 times by upregulating the expression of aroF. The application of CM in salinized soil leads to an obvious enrichment in many pathways in *B. campestris*. Among them, in the amino sugar and nucleotide sugar metabolism pathway, the application of CM lead to the downregulation of the expression of GDP-L-Fucose by upregulating the expression of GPMM and downregulating the expression of PMM, and the expression of N-carbamoyl-L-aspartate was downregulated by 1.8 times by upregulating the expression of GFPT. In the Phenylalanine metabolism pathway, the expression of N-Acetyl-L-phenylalanine was upregulated by 2.36 times. In the alpha-Linolenic acid metabolism pathway, the expression of 9(S)-HOTrE was upregulated by 0.62 times by upregulating the expression of LOX2S, and the expression of traumatic acid was downregulated by 2.63 times by downregulating the expression of HPL1. After the application of CM, the number of pathways enriched was less in Na_2_CO_3_ treatments than in NaCl treatments. After the application of CM, the expression of oxoglutaric acid was downregulated by 2.8 times by upregulating the expression of IDH3 in the citrate cycle (TCA cycle) pathway, the expression of fumaric acid was upregulated by 0.76 times by downregulating the expression of SDHB, and the expression of (S)-Malate (L-Malic acid) was upregulated by 0.58 times by downregulating the expression of FH. Similar to NaCl treatments, there were also changes in the enrichment of DEGs and DEMs in the alanine, aspartate, and glutamate metabolism pathway in Na_2_CO_3_ treatments, in which the expression of n-carbamoyl-l-aspartate was downregulated by 1.18 times. Different from NaCl treatments, the biosynthesis of other secondary metabolites pathway was mainly enriched for the DEGs and DEMs in Na_2_CO_3_ treatments. Among them, the expression of sinapic acid in phenylpropanoid biosynthesis pathway was downregulated by 2.69 times, and the expression of neohesperidin in flavonoid biosynthesis pathway was upregulated by 2.38 times, compared with those in the YCK treatment.

#### Combined Analysis of Dry Weight, Physiological Indexes, Ion Contents, Transcriptomics, and Metabolomics in *B. campestris*

**Figure 6:**
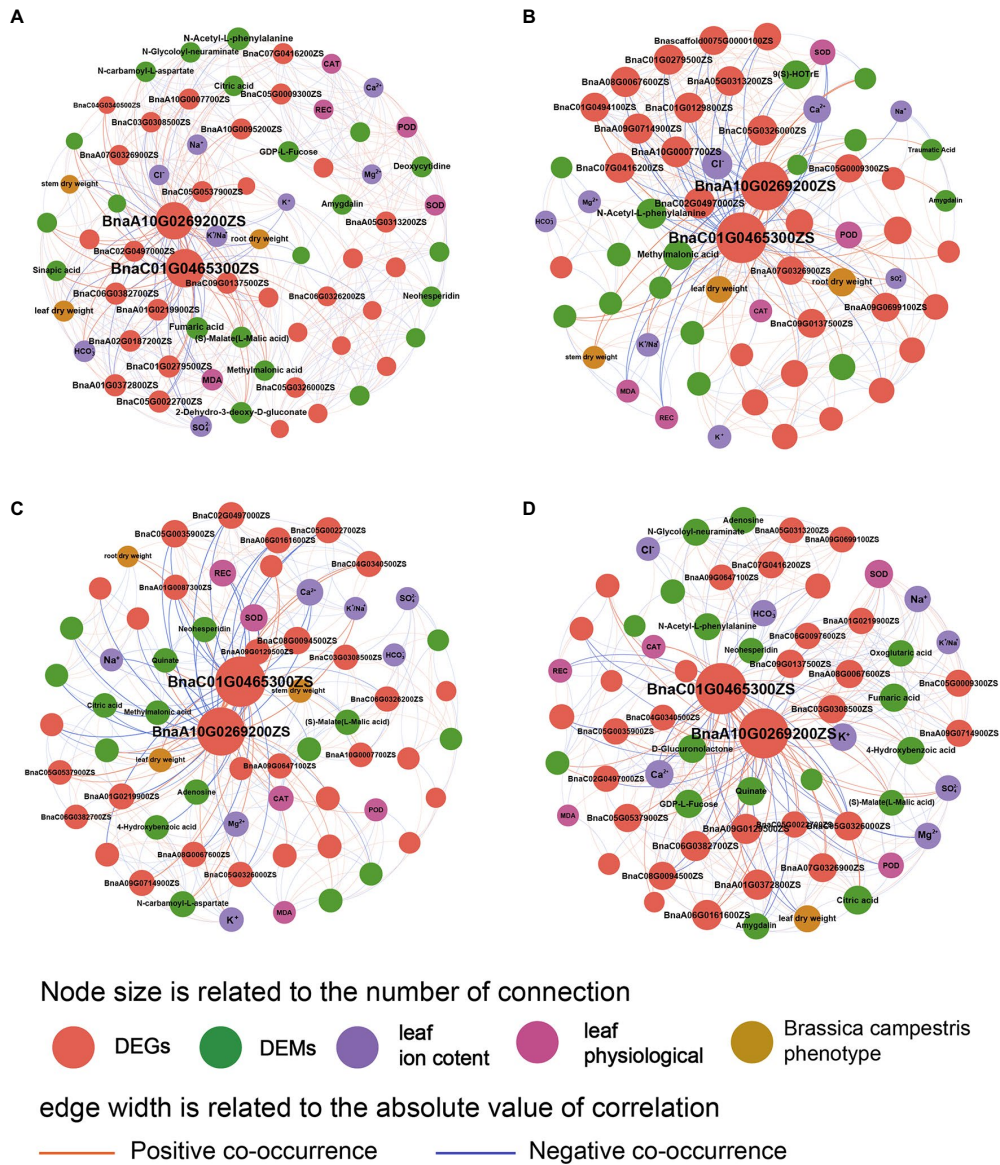
Co-occurrence network of phenotype, physiological indexes, ion contents, differential expressed genes, and differential expressed metabolites of *Brassica campestris.* **(A)** YCK treatment, **(B)** JCK treatment, **(C)** YP treatment, and **(D)** JP treatment. There are positive or negative correlations between the two indexes connected (*p* < 0.05). The indexes with connectivity greater than 15 are marked with words.

The network diagram showed the relationships among various indexes of *B. campestris* (Figure 6). It was found that the regulation mechanism in *B. campestris* leaves varied in different treatments. The DEGs (*BnaA10G0269200ZS* and *BnaC01G0465300ZS*) had good interactions with other indexes in different treatments. Ion contents had greater effects on *B. campestris* in NaCl treatments (YCK and YP treatment) than in Na_2_CO_3_ treatments (JCK and JP treatment; Figure 6A), which was mainly reflected in the effects of K^+^, Na^+^, Cl^−^, and K^+^/Na^+^ ratio on ion homeostasis in *B. campestris*. The phenotype and ion contents of *B. campestris* were directly regulated by genes, and there were also ways of mutual regulations among ion contents, metabolites, and physiological indexes. The main regulatory genes (*BnaA10G0269200ZS* and *BnaC01G0465300ZS*) directly affected the phenotypic indexes, and the effect on root dry weight was greater than that on leaf and stem dry weight. Moreover, the regulation on root and leaf dry weight was negative. The regulatory effects on DEGs (*BnaA10G0269200ZS* and *BnaC01G0465300ZS*) were greater in Na_2_CO_3_ treatments (JCK and JP treatment) than in NaCl treatments (YCK and YP treatment; Figure 6B). The content of Cl^−^ was negatively correlated with many genes. The dry weight of root, leaves, and stem were positively correlated with the expression of *BnaA10G0269200ZS* and *BnaC01G0465300ZS*, and showed a trend of leaf dry weight > root dry weight > stem dry weight. The stem dry weight was positively correlated with K^+^/Na^+^ ratio. BnaC01G0465300ZS was positively correlated with methylmalonic acid and POD, and negatively correlated with CAT and N-Acetyl-L-phenylalanine.

After the application of CM, the *BnaA10G0269200ZS* and *BnaC01G0465300ZS* (DEGs) were still the main factors in the regulation. The responses of *B. campestris* to saline and alkaline stresses changed, showing that the interactions between genes and metabolites were strengthened and the interactions between genes and ions were weakened. In NaCl treatments (YCK and YP treatment), the DEGs positively regulated the stem and leaf dry weight by regulating the ion balance, and most of DEGs showed a positive correlation with POD and CAT, and a negative correlation with REC (Figure 6C). At the same time, the interactions between the DEMs (Neohesperidin, Quinate, and Adenosine) and the DEGs (*BnaA10G0269200ZS* and *BnaC01G0465300ZS*) were strengthened. In Na_2_CO_3_ treatments (JCK and JP treatment), after the application of CM, more metabolites were involved in the regulation of alkaline stress, and the DEMs (D-Glucuronolactone, GDP-L-Fucose, and Quinate) were positively regulated (Figure 6D). Among them, BnaA10G0269200ZS was positively correlated with Ca^2+^ and K^+^, and BnaC01G0465300ZS and many DEGs were negatively correlated with ${\mathrm{HCO}}_{3}^{-}$ and positively correlated with CAT. Moreover, the application of CM had the greatest effects on the leaf dry weight. It was correlated with Amygdalin and negatively correlated with *BnaA07G0326900ZS, BnaA01G0372800ZS*, and citric acid.

## Discussion

Our results showed that there were similarities and differences in phenotypic plasticity and genetic adaptability of plants under saline and alkaline stresses. The toxicity of saline and alkaline stresses to plants and the induced self-regulation mechanism of plants were also different. Compare with the saline stress, Alkaline stress had greater ion toxicity and oxidative stress, which is reflected in higher Na^+^, CAT, and MDA contents (Fang et al., 2021). This is because high pH stress and ion imbalance may occur in addition to the ion toxicity caused by Na^+^ when plants are under alkaline stress (Han et al., 2018). Excessive Na^+^ will make a competitive relationship with K^+^, Ca^2+^, and Mg^2+^, which break the ion balance in plants, while high pH will increase cellular oxidative stress and cause massive precipitation of crucial mineral elements, such as phosphorus, calcium, and magnesium, eventually leading to nutrient deficiency and biomass decline in plants (Tahkokorpi et al., 2012; Wang et al., 2015). In this study, the network diagram showed that the detoxification of antioxidant enzymes was suppressed under saline and alkaline stresses, resulting in the accumulation of reactive oxygen species in cells (Figure 6), which seriously destroys the cell structure and macromolecules, such as DNA, lipids, and enzymes (Liu et al., 2021). It is indicated that in severe saline and alkaline soils, the ion imbalance in soils could affect the ion balance in plants and make plants alter apparent plasticity to response the saline and alkaline stress environment (Yang et al., 2018; Lamers et al., 2020). Combined with transcriptional and metabolomics analysis of self-genetic adaptation of *B. campestris*, we found that *BnaA10G0269200ZS* and *BnaC01G0465300ZS* were the main regulators on biomass, ion balance, and antioxidant enzyme activities of *B. campestris.* In this research, the osmotic protection function of *B. campestris* is regulated by regulating metabolites including fumaric acid, (S)-L-Malic acid, and methylmalonic acid, to improve the tolerance to saline and alkaline stresses and maintain cellular water balance (Talaat et al., 2015). Under saline stress, the content of sinapic acid is regulated to maintain cell water balance, to cope with the osmotic stress caused by salt (Liu et al., 2014). However, under alkaline stress, the content of N-Acetyl-L-phenylalanine is regulated to cope with the osmotic stress caused by alkali.

Application of compound material altered the phenotypic plasticity and genetic adaptation of *B. campestris* under saline and alkaline stresses. The phenotypic analysis showed that the effect of compound material application on the growth of *B. campestris* was significantly different from that of the CK (YCK & JCK) treatments. The compound material application increased K^+^ and Mg^2+^ in leaves, but decreased Na^+^ and REC content to maintain cellular ion homeostasis. Besides, the activities of CAT and POD in antioxidant system were increased and the ionic and osmotic homeostasis of leaf cells were rebuilt to improve the tolerance to saline and alkaline stresses, which eventually increased the dry weight of various organs of *B. campestris*. Genetic adaptation is reflected in gene modification, including gene expression and quantitative change (Wu et al., 2021). Transcriptomic analysis showed that the application of CM changed the expression of genes in *B. campestris* under saline stress, such as the expression of genes encoding K^+^ and Na^+^ transporters and related enzyme activities [such as *BnaA09G0714900ZS*, *BnaC03G0308500ZS*, and *BnaA09G0647100ZS* (Gu et al., 2021)]. Previous study has found that the increased stress tolerance of transgenic plants may be due to the upregulation of genes involved in protecting intracellular K^+^ and Na^+^ homeostasis from damage (Meng et al., 2020). In this study, the expression of genes related to potassium ion transport and regulation of potassium ion transport and superoxide dismutase were upregulated. The regulation of these genes is helpful for *B. campestris* to improve the tolerance to saline and alkaline stresses (Zhang et al., 2021). The multiomics analysis found that the application of CM changed amino acid metabolism pathways, including Alanine, aspartate and glutamate metabolism, Phenylalanine metabolism, and Tyrosine metabolism by regulating the transcription factors (*IDH3, HPD,* and *pel*). Amino acids play an important role in promoting plant growth and resisting various abiotic stresses (Widodo et al., 2009; Caldana et al., 2011). This study found that compound material application could promote amino acid synthesis in the leaves. This plays a role in maintaining cell structure and promoting crop growth (Li and Song, 2019), and also improves the osmotic pressure and antioxidant properties of cells (Jia et al., 2020). Therefore, it was inferred that the application of CM could reconstruct ionic homeostasis and osmotic homeostasis under saline stress, to improve the tolerance of *B. campestris* to saline stress. However, the application of CM had different regulatory effects on *B. campestris* leaves under saline stress and alkaline stress. Compared with the salt control treatment (YCK), the application of CM changed the metabolic pathways related to organic acid metabolism by regulating the expression of transcription factors (*IDH3, HPDG, PMM,* and *GFPT*), such as phenylalanine metabolism and alpha-Linolenic acid metabolism. It indicates that under saline stress, the compound material could enable *B. campestris* to synthesize a large amount of organic acids, which not only enhances the antioxidant system, but also promotes the synthesis of important structural substances in plant cells (You et al., 2019). What’s more, the application of CM also changed the biosynthetic pathways of other secondary metabolites under alkaline stress by regulating transcription factors (*PAL, CYP37A*), including phenylpropanoid biosynthesis and flavonoid biosynthesis pathway. Study has found that Phenylpropane secondary metabolism pathway affects the synthesis of lignin, flavonoids, and other secondary substances (Chun et al., 2019). The biosynthesis of phenylpropane is related to the synthesis of flavonoids and lignin, which could improve the osmotic regulation and enhance the tolerance of *Fraxinus mandshurica* to stress (Sharma et al., 2019), which indicated that the application of CM might improve the osmotic regulation by upregulating the secondary metabolites content in *B. campestris*, to enhance the tolerance to saline and alkaline stresses.

Our results showed that there was difference between the self-regulation mechanism of *B. campestris* in response to saline and alkaline stresses and the regulation mechanism of compound material (Figure 6). From the perspective of apparent plasticity, the growth inhibition of *B. campestris* under saline and alkaline stresses was alleviated by the application of CM. However, there was no difference in the effect of the application of CM on the biomass of *B. campestris* under saline and alkaline stresses. The application of CM helped achieve the ion homeostasis in *B. campestris under saline and* alkaline stress. Under saline stress by increasing the content of K^+^, Ca^2+^, Mg^2+^, ${\mathrm{SO}}_{4}^{2-}$, and ${\mathrm{HCO}}_{3}^{-}$ and reducing that of Na^+^, and it helped achieve the ion homeostasis in *B. campestris*, thus alleviating the ion toxicity (Ma et al., 2019), under alkaline stress by increasing the content of Ca^2+^ and K^+^ and reducing that of Cl^−^ and ${\mathrm{HCO}}_{3}^{-}$. At the same time, the application of CM improved the detoxification of antioxidant enzymes, which is reflected in the regulation of SOD, CAT, and MDA. This research antioxidant enzymes can scavenge reactive oxygen species in cells, thereby reducing oxidative stress under saline and alkaline stresses (Lu et al., 2021). We also found that in this study, the application of CM made more metabolites participate in the responses of *B. campestris* to alkaline stress, in which the DEMs including D-Glucuronolactone, GDP-L-Fucose, and Quinate were positively regulated by DEGs (*BnaA10G0269200ZS, BnaC01G0465300ZS*). Previous study has found that GDP-L-Fucose and Quinate obviously accumulate in leaves when plants are short of boron for a long time (Liu et al., 2015). Therefore, the application of CM might promote the normal operation of carbohydrates by regulating the boron content in *B. campestris*, to enhance the tolerance to saline and alkaline stresses. The citric acid and adenosine regulated under saline stress and the neohesperidin and fumaric acid regulated under alkaline stress play an important role in plant stress resistance by maintaining cellular osmotic pressure and ionic homeostasis (Chen et al., 2016).

## Conclusion

**Figure 7:**
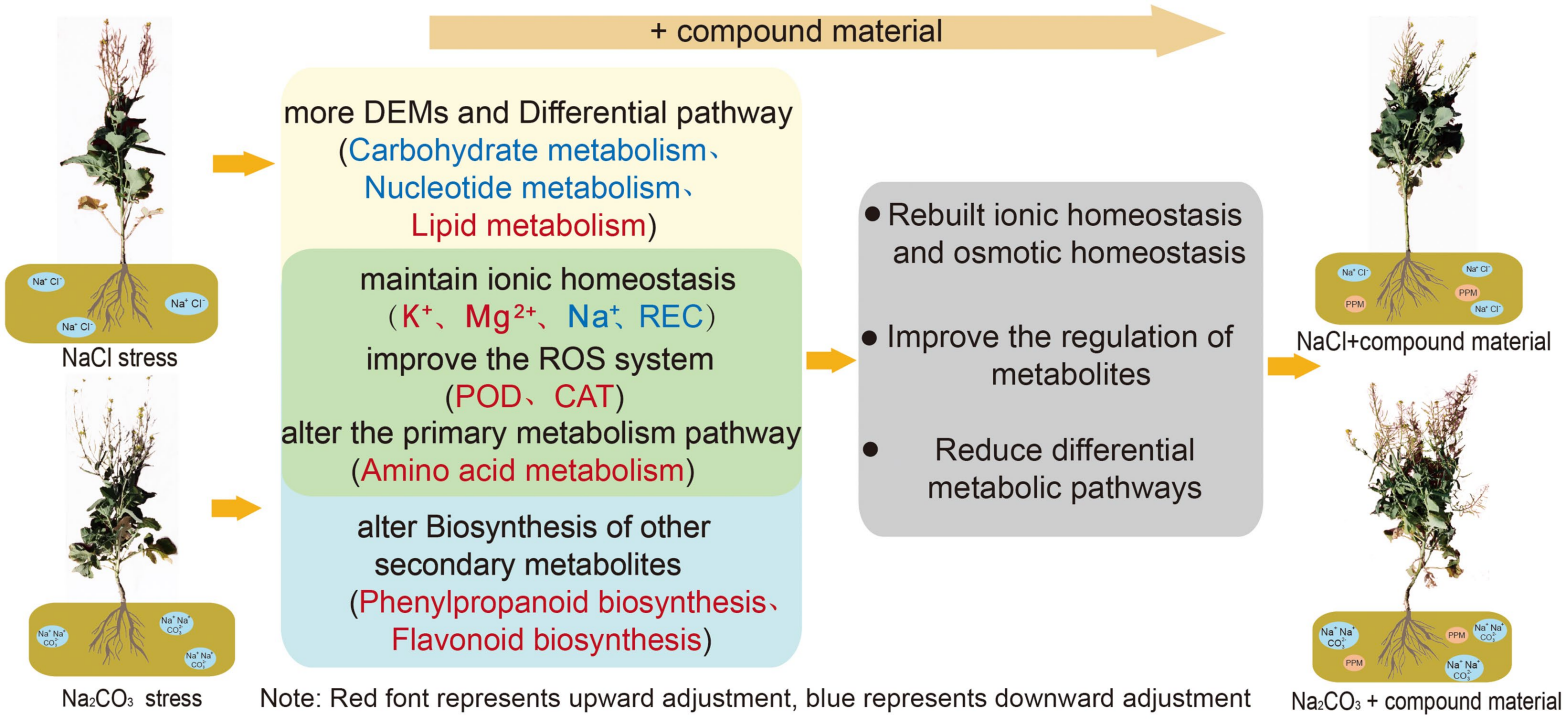
Regulation mechanisms of compound material on the responses of *Brassica campestris* of compound material to saline and alkaline stresses of *Brassica campestris*. Yellow and blue represent the regulation mechanisms of the responses to saline stress and alkaline stress, respectively, and green represents the common regulation mechanism of the responses to saline stress and alkaline stress. Red indicates upregulation, and blue indicates downregulation.

Saline stress and alkaline stress had different degrees of harm to oilseed rape (Figure 7). Alkaline stress had greater ion toxicity to oilseed rape through the increase of Na^+^ content and the decrease of K^+^/Na^+^ ratio. The application of CM could recover the ionic homeostasis and osmotic homeostasis in leaves through genetic adaptation and phenotypic plasticity. The CM could increase K^+^ content and K^+^/Na^+^ ratio in leaves by upregulating genes related to the regulation of potassium ion transport in saline and alkaline soils, and change the amino acid metabolic pathway through upregulating transcription factor IDH3 and HPD and downregulating the expression of pel, which increased the activities of SOD, POD, and CAT and decreased the contents of Na^+^, MDA, and REC in oilseed rape leaves. However, there were differences in the regulation mechanism of CM on oilseed rape in saline and alkaline soil. In saline soil, the CM regulated nucleotide metabolism and lipid metabolism through the upregulation of transcription factors GPMM and GFPT and the downregulation of glna and PMM, to improve the antioxidant capacity of oilseed rape. In alkaline soil, the CMl regulated the metabolic pathway of secondary metabolites by upregulating the transcription factors PAL and CYP73A to improve the osmotic regulation of oilseed rape. Moreover, the application of CM made more metabolites participate in the process of regulating ion balance to improve the tolerance to saline and alkaline stresses. This study showed that the application of CM could improve the tolerance of oilseed rape to saline and alkaline stresses and play a better regulatory role in saline soil. Our findings are helpful to develop a practical method for sustainable agriculture in saline and alkaline soils.

## Data Availability Statement

The original contributions presented in the study are publicly available. These data can be found at: National Center for Biotechnology Information (NCBI) BioProject database under accession number PRJNA772052.

## Author Contributions

ZL designed the study. ZL and DH collected the data. ZL and MA wrote the paper with input from all authors. All authors contributed to the final version of the manuscript.

## Funding

The research was supported by the Major Science and Technology Project of Xinjiang Production and Construction Corps, China (2018AA005); National Key Research and Development Program of China, China (2016YFC0501406); and National Natural Science Foundation of Xinjiang Province, China (31860591).

## Conflict of Interest

The authors declare that the research was conducted in the absence of any commercial or financial relationships that could be construed as a potential conflict of interest.

## Publisher’s Note

All claims expressed in this article are solely those of the authors and do not necessarily represent those of their affiliated organizations, or those of the publisher, the editors and the reviewers. Any product that may be evaluated in this article, or claim that may be made by its manufacturer, is not guaranteed or endorsed by the publisher.

## Footnotes

^1^ www.biocloud.net

^3^ https://www.rproject.org/

^4^ https://gephi.org/
