# Supplementary material for: Transcriptomic and Metabolomic Analyses Reveal the Differential Regulatory Mechanisms of Compound Material on the Responses of Brassica campestris to Saline and Alkaline Stresses
Source: Front Plant Sci. 2022 Feb 23;13:820540. doi: 10.3389/fpls.2022.820540 (PMC8905141; doi:10.3389/fpls.2022.820540)
Supplement: Supplementary file 1 [file Data_Sheet_1.docx]

Supplementary Material

# Supplementary Data

The raw reads of  Brassica napus Transcriptome or Gene expression (TaxID: 3708) are available under accession PRJNA772052 at NCBI Sequence Read Archive (SRA) repository. All data has been released

# Supplementary Figures and Tables

##
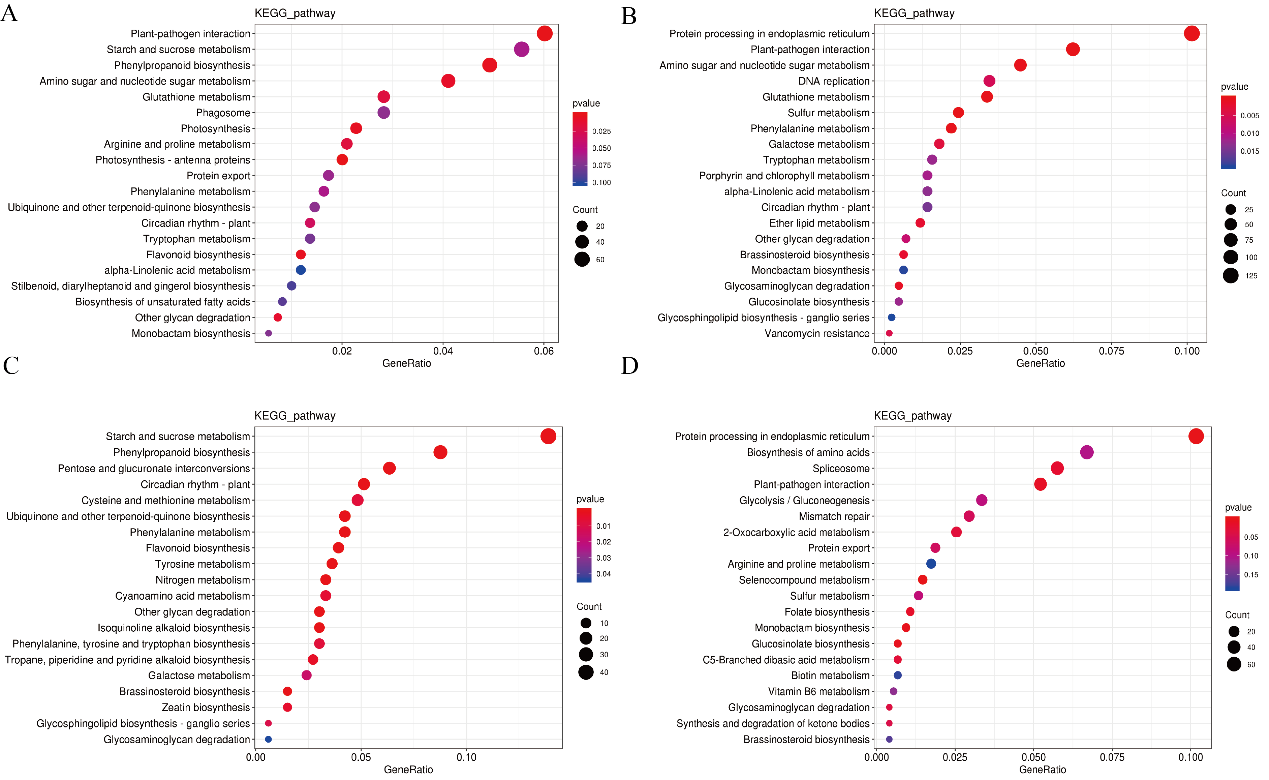
Supplementary Figures

**Supplementary Figure 1.** KEGG enrichment analysis. The top 20 enriched KEGG pathways in NaCl treatments (YCK and YP treatment) (a). The top 20 enriched KEGG pathways in Na_2_CO_3_ treatments (JCK and JP treatment) (b). The top 20 enriched KEGG pathways in the control (YCK and JCK treatment) (c). The top 20 enriched KEGG pathways in compound material treatments (YP and JP treatment).


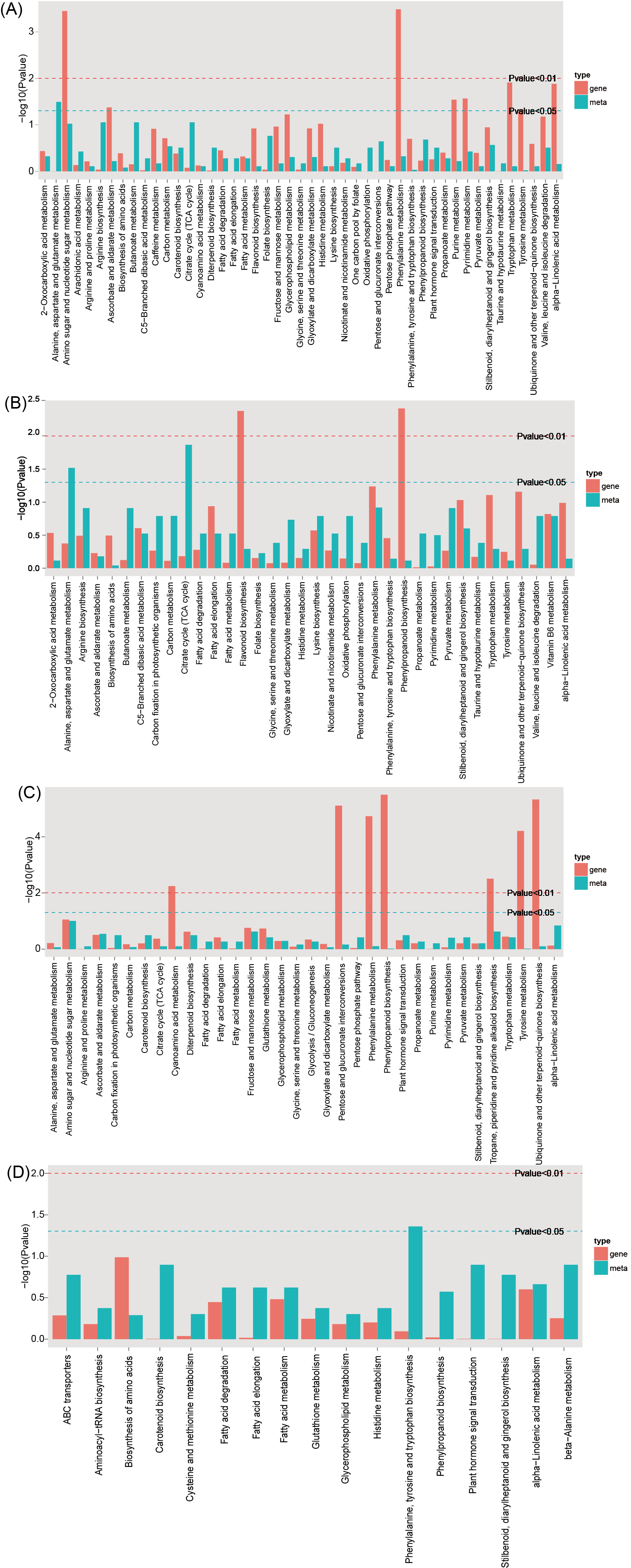


##
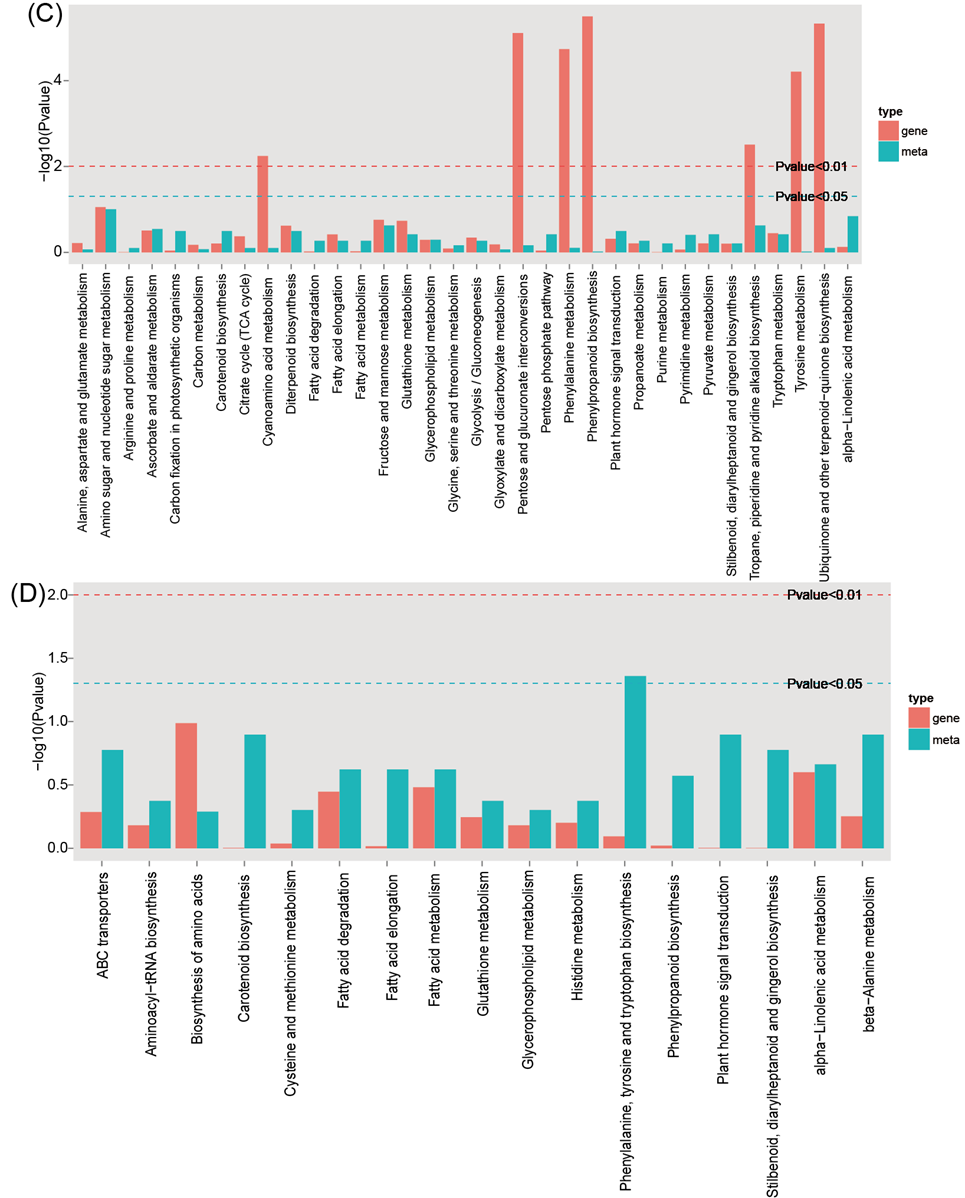


**Supplementary Figure 2.** P-value histogram of KEGG enrichment analysis for integrating metabolomics and transcriptomics. NaCl treatments (YCK and YP treatment) (a); Na_2_CO_3_ treatments (JCK and JP treatment) (b); Control (YCK and JCK treatment) (c); Compound material treatments (YP and JP treatment) (d). The x-axis represents the enriched metabolic pathways, and the y-axis represents - log (P value). Red represents the P value for the enrichment of differentially expressed genes in pathways, and green represents the P value for the enrichment of differentially expressed metabolites in pathways.

## Supplementary Tables

**Supplementary Table 1.** Sequencing data statistics

| **Samples** | **Clean reads** | **Clean bases** | **GC Content** | **%≥Q30** |
| --- | --- | --- | --- | --- |
| J-CK-1 | 22,202,595 | 6,639,744,542 | 45.55% | 94.73% |
| J-CK-2 | 19,737,079 | 5,892,253,674 | 45.96% | 94.73% |
| J-CK-3 | 19,235,403 | 5,751,696,190 | 45.58% | 94.48% |
| J-P-1 | 21,008,391 | 6,277,966,856 | 46.16% | 94.21% |
| J-P-2 | 23,104,811 | 6,915,006,624 | 46.27% | 94.20% |
| J-P-3 | 19,845,191 | 5,945,722,740 | 45.35% | 93.40% |
| Y-CK-1 | 25,951,843 | 7,771,135,320 | 45.21% | 94.31% |
| Y-CK-2 | 22,038,105 | 6,586,437,488 | 45.35% | 94.41% |
| Y-CK-3 | 25,331,915 | 7,578,677,336 | 45.59% | 94.43% |
| Y-P-1 | 21,310,491 | 6,382,574,596 | 45.55% | 94.22% |
| Y-P-2 | 20,910,451 | 6,255,174,948 | 46.40% | 94.42% |
| Y-P-3 | 20,782,904 | 6,218,665,516 | 45.54% | 94.30% |

Note: (1) Samples: Sample name; 
(2) Clean reads: Counts of clean PE (pair-end) reads;

(3) Clean bases: Total base number of clean data; 
(4) GC content: Percentage of G (Guanine)and C (Cytosine) in clean data; 
(5) ≥ Q30%: Percentage of bases with Q-score no less than Q30.
